# Preparation of Zirconia Nanofibers by Electrospinning and Calcination with Zirconium Acetylacetonate as Precursor

**DOI:** 10.3390/polym11061067

**Published:** 2019-06-20

**Authors:** Vyacheslav V. Rodaev, Svetlana S. Razlivalova, Andrey O. Zhigachev, Vladimir M. Vasyukov, Yuri I. Golovin

**Affiliations:** Institute for Nanotechnology and Nanomaterials, Derzhavin Tambov State University, Internatsionalnaya str. 33, 392000 Tambov, Russia; razlivalova8@yandex.ru (S.S.R.); andreyzhig2009@yandex.ru (A.O.Z.); space-1985@mail.ru (V.M.V.); golovin@tsu.tmb.ru (Y.I.G.)

**Keywords:** electrospinning, ceramic nanofibers, ZrO_2_, morphological evolution, support for catalysts

## Abstract

For the first time, zirconia nanofibers with an average diameter of about 75 nm have been fabricated by calcination of electrospun zirconium acetylacetonate/polyacrylonitrile fibers in the range of 500–1100 °C. Composite and ceramic filaments have been characterized by scanning electron microscopy, thermogravimetric analysis, nitrogen adsorption analysis, energy-dispersive X-ray spectroscopy, and X-ray diffractometry. The stages of the transition of zirconium acetylacetonate to zirconia have been revealed. It has been found out that a rise in calcination temperature from 500 to 1100 °C induces transformation of mesoporous tetragonal zirconia nanofibers with a high specific surface area (102.3 m^2^/g) to non-porous monoclinic zirconia nanofibers of almost the same diameter with a low value of specific surface area (8.3 m^2^/g). The tetragonal zirconia nanofibers with high specific surface area prepared at 500 °C can be considered, for instance, as promising supports for heterogeneous catalysts, enhancing their activity.

## 1. Introduction

Nanofibers are fibers with diameters in the nanometer range. They attract a great deal of attention due to their remarkable properties compared to their microfiber counterparts, namely high surface area, high surface-to-mass ratio, interconnected porous structure, and flexibility in surface functionalization [1].

Among a number of nanofiber fabrication techniques, electrospinning is the most commonly used method due to its simplicity, adaptability, cost-efficiency, and versatility, as well as the capability to control the diameter, composition, and morphology of filaments. Besides, electrospinning is the only method for the large-scale fabrication of nanofibers [1].

Electrospinning can be used for the production of ceramic nanofibers. This process includes the following stages: Preparation of a spinnable solution containing the ceramic precursor and a binding polymer; spinning of the prepared solution; calcination of the electrospun composite fibers to remove unwanted constituents and to obtain the desired ceramic phase; and finally, sintering at an elevated temperature to fabricate the final ceramic nanofibers with the required structure [2].

Among ceramic nanofibers, zirconia nanofibers have significant potential to be utilized in a variety of applications because of their high chemical and thermal stability, high ionic conductivity, biocompatibility, and polymorphism of ZrO_2_ [3]. For example, zirconia nanofibers exhibit phase-dependent catalytic activity and selectivity [4,5], improve performance of solid oxide fuel cells [6,7], increase the mechanical properties of composites [8,9], and act as flexible scaffolds for bone tissue regeneration [10] and as supports for functional nanoparticles [11,12].

Due to its availability and reasonable price, zirconium oxychloride is frequently used as a ceramic precursor in the zirconia nanofiber fabrication process [13,14,15,16]. Its main disadvantage is that the oxychloride hydrolysis is not fast enough and still continues even after the electrospinning solution has been spun to form composite fibers. Long-term hydrolysis may negatively affect as-spun filament morphology. On the other hand, rapid hydrolysis and gelation rates may lead to blockage of the spinneret, preventing the electrospinning process. To ensure continuity of the electrospinning process and homogeneous and defect-free composite fiber formation, it is necessary to precisely select the kinds of the precursor and the solvent, the concentrations of the precursor and the polymer, and the special additives stabilizing the precursor and facilitating the electrospinning process [17]. In [5,18,19], zirconium acetate was chosen as the precursor to fabricate zirconia nanofibers, and acetic acid was used as a stabilizing additive.

Poly(vinyl pyrolidone) (PVP) is the most popular polymer employed as a matrix due to its high solubility in ethanol, water, and dimethylformamide (DMF), and its good compatibility with many metal alkoxides and salts [17]. Using DMF as a solvent minimizes hydrolysis and gelation of the ceramic precursor without adding any stabilizing additives [20]. In spinning DMF-based solutions, PVP can be replaced with polyacrylonitrile (PAN), which retains shape stability at higher temperatures than PVP. Besides, in the case of DMF, prepared electrospun PAN nanofiber mats are mechanically stronger than the corresponding electrospun PVP nanofiber mats [21,22]. This is expected to facilitate preparatory work with as-spun composite fiber mats before their further processing.

Zirconium acetylacetonate (Zr(C_5_H_7_O_2_)_4_) (hereinafter referred to as ZrAA) is easily dissolvable in DMF and can be considered as an alternative to the above-mentioned zirconia precursors (zirconium oxychloride and zirconium acetate). In [20,23], similar to zirconium acetylacetonate, aluminum acetylacetonate was successfully used for alumina nanofiber fabrication via electrospinning of aluminum acetylacetonate/PAN/DMF solutions. Alumina nanofibers produced in this way possessed extremely high tensile strength, with a value of 11.4 ± 1.1 GPa [23].

This paper for the first time reports zirconia nanofibers fabricated from electrospun composite ZrAA/PAN fibers, and the results of the comprehensive investigation of the filaments’ morphological evolution during heat treatment that is important for their practical use.

## 2. Materials and Methods

A 10 wt. % polymer solution was prepared by adding 1 g PAN (molecular weight Mw = 150,000; Sigma-Aldrich, Saint Louis, MO, USA) into 9 g DMF, with periodic stirring by hand for 2 h at 50 °C. Then, 0.3 g ZrAA (Sigma-Aldrich, Saint Louis, MO, USA) was added into the solution under stirring in the ultrasonic bath for 6 min at 42 kHz at room temperature to prepare the transparent electrospinning solution.

The prepared composite solution was transferred into a 10 ml plastic syringe and electrospun using the electrospinning machine NANON-01A (MECC, Fukuoka, Japan) at a feeding rate of 1.2 mL/h through a 21 G blunt-tip needle. The following operative parameters were chosen to fabricate smooth and bead-free composite fibers: The accelerating voltage was 23 kV, and the distance between the needle tip and the flat collector covered by aluminum foil was 21 cm. The fibers were collected in the form of non-woven mats.

As-spun mats were calcined at different temperatures in the range of 500–1300 °C for 1 h using two-stage heating: Heating to 500 °C with a heating rate of 1 °C/min, and then further heating to the target temperature with a heating rate of 5 °C/min. To prevent fiber destruction, a low heating rate at the first annealing stage was chosen to ensure delicate removal of the decomposition products of the ceramic precursor and binding polymer.

The surface texture, diameter, and elemental composition of the fibers were analyzed by the scanning electron microscope (SEM) Merlin (Carl Zeiss, Oberkochen, Germany) coupled with an energy-dispersive X-ray spectrometer (EDS) INCA Energy 350X-Max 80 (Oxford Instruments, Abingdon, UK). XRD patterns were recorded in the 20–80° 2θ range by the X-ray diffractometer (XRD) D2 Phaser (Bruker AXS, Karlsruhe, Germany) using CuKα1 monochromatic radiation. XRD patterns were assigned using the PDF-2 Diffraction Database File compiled by the International Centre for Diffraction. The phase content was determined from XRD patterns by the Rietveld method in the TOPAS software (Bruker AXS, Karlsruhe, Germany). SEM, EDS, and XRD measurements were carried out at room temperature. Nitrogen adsorption–desorption isotherms at 77 K were registered with a gas sorption analyzer Autosorb iQ-C (Quantachrome Instruments, Boynton Beach, FL, USA). Specific surface areas of the nanofibers were determined with the Brunauer–Emmett–Teller (BET) method. Total pore volume was calculated through the quantity of adsorbed N_2_ at the relative pressure of 0.995. The thermogravimetric (TG) analysis was performed on the thermal analyzer EXSTAR TG/DTA7200 (SII Nano Technology, Tokyo, Japan) in air atmosphere with a heating rate of 10 °C/min.

## 3. Results and Discussion

**Figure 1:**
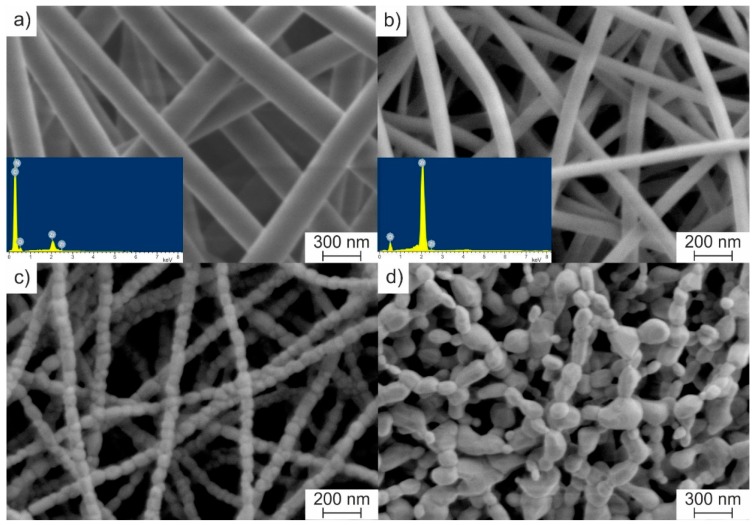
SEM images of zirconium acetylacetonate/polyacrylonitrile (ZrAA/PAN) fibers (**a**) as-spun, and calcined at (**b**) 500, (**c**) 1100, and (**d**) 1300 °C. The inserts show their energy-dispersive X-ray spectrometer (EDS) spectra at appropriate temperatures.

The electrospinning process of the prepared solution resulted in randomly distributed cylindrical bead-free ZrAA/PAN fibers with smooth surfaces (Figure 1a). Fiber diameters ranged from 230 to 330 nm. The average diameter was 275 ± 19 nm.

The obtained EDS data indicate that the prepared fibers, as the solution, contain the binding polymer (C, N) and zirconia precursor (Zr, C, O) (insert of Figure 1a). The heat treatment leads to a decrease in fiber diameter. The average diameter of calcined fibers at 500 °C is 86 ± 7 nm (Figure 1b). According to the EDS data, ZrAA/PAN fibers calcined at 500 °C are zirconia nanofibers (insert of Figure 1b). An increase in calcination temperature from 500 to 800 °C results in a slight decrease in the average diameter of ZrO_2_ nanofibers to 71 ± 6 nm, due to their sintering. Within the measurement error, no further filament shrinkage is observed in the range of 800–1100 °C. At 1100 °C, the average diameter of ceramic nanofibers is 70 ± 3 nm, and zirconia grains attain a size equal to the fiber diameter (Figure 1c). A calcination temperature increase to 1300 °C essentially enhances diffusion, which leads to ZrO_2_ nanofiber destruction (Figure 1d).

**Figure 2:**
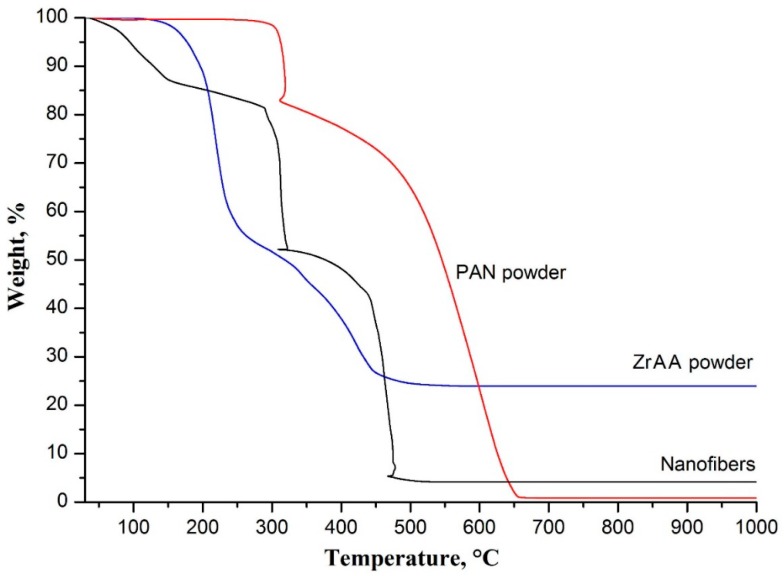
Thermogravimetric (TG) curves of electrospun ZrAA/PAN fibers and powders of initial components.

The decreases in composite fiber diameter correspond to the weight changes determined by the TG analysis. The obtained TG curves of as-spun ZrAA/PAN fibers, and initial ZrAA and PAN powders are shown in Figure 2.

The TG curve of as-spun ZrAA/PAN fibers is characterized by several weight losses. The first weight loss before 165 °C results from the removal of the residual solvent from the composite fibers, since it is not observed in the TG curves of ZrAA and PAN. The second one occurs in the range of 165–290 °C and is related to Zr(OH)(CH_3_COO)_3_ formation, which is confirmed by the observed weight loss of about 42% at 290 °C in the TG curve of ZrAA. The next weight loss before 325 °C is mainly attributed to oxidative stabilization of PAN. During the stabilization process, the cyclization of the nitrile groups and cross-linking of the chain molecules are followed by dehydrogenation [24]. Besides, Zr(OH)(CH_3_COO)_3_ decomposition leading to ZrO(CH_3_COO)_2_ occurs in the range of 290–355 °C (with a weight loss of about 54% at 355 °C in the TG curve of ZrAA). This explains the further weight drop in the TG curve of filaments as the temperature rises above 325 °C. The final weight loss in the TG curve of fibers, observed in the range of 355–500 °C, is associated with polymer combustion and ZrO_2_ formation (with a 75% weight loss at 500 °C in the TG curve of ZrAA). So, we can conclude that the transformation process of ZrAA to ZrO_2_ goes through the following stages: ZrAA → Zr(OH)(CH_3_COO)_3_ → ZrO(CH_3_COO)_2_ → ZrO_2_.

**Figure 3:**
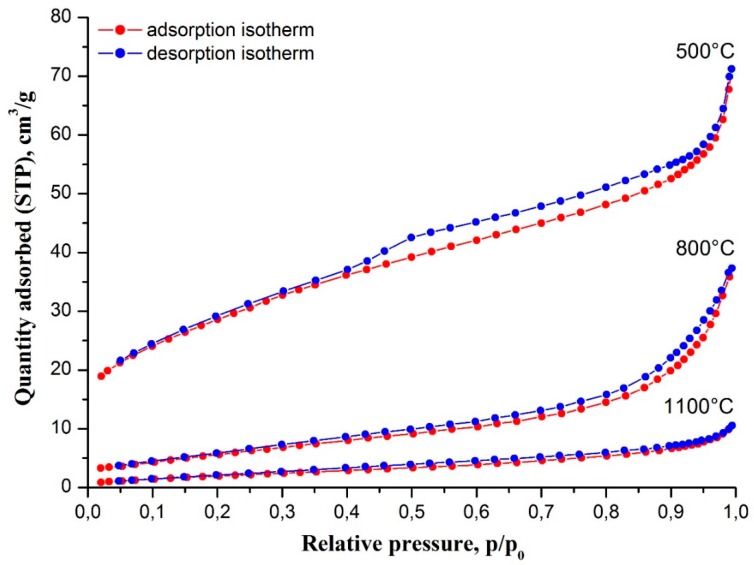
Nitrogen adsorption–desorption isotherms at 77 K of electrospun ZrAA/PAN fibers calcined at different temperatures.

The rise in filament calcination temperature also affects their specific surface area and porosity. Figure 3 shows nitrogen adsorption–desorption isotherms at 77 K of zirconia nanofibers calcined at different temperatures. According to the International Union of Pure and Applied Chemistry (IUPAC) classification, ceramic nanofibers prepared at 500 °C show isotherms of type IV with a hysteresis loop of H4 type, which is associated with capillary condensation in narrow slit-like mesopores. In our case, slit-like mesopores are most likely formed by the boundaries of adjacent ZrO_2_ grains. Their appearance is a result of ZrAA and PAN decomposition. The total volume of mesopores was determined as 0.110 cm^3^/g. The specific surface area of the filaments calcined at 500 °C is 102.3 m^2^/g. Zirconia nanofibers prepared at 800 and 1100 °C showed isotherms of type II inherent to low porous or non-porous materials. The increase in calcination temperature leads to filament porosity reduction and decreases in their specific surface area: 0.058 cm^3^/g and 22.5 m^2^/g, respectively, at 800 °C; and 0.016 cm^3^/g and 8.3 m^2^/g, respectively, at 1100 °C, due to sintering.

**Figure 4:**
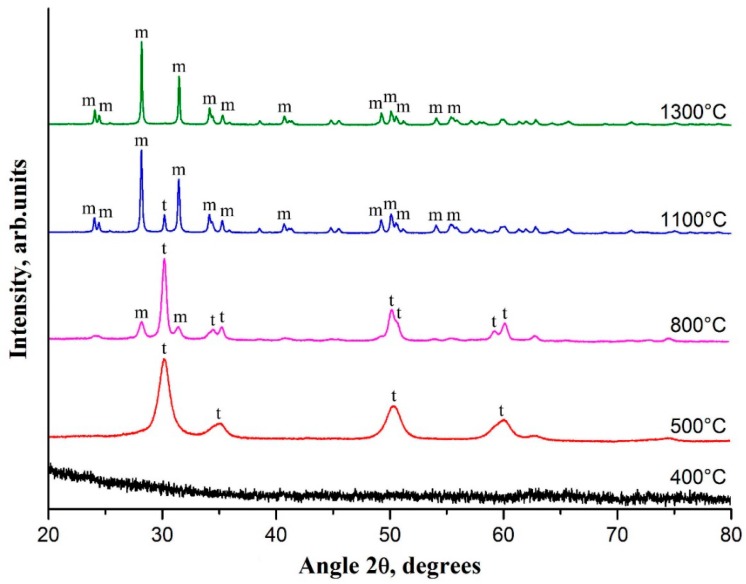
XRD patterns of electrospun ZrAA/PAN fibers calcined at different temperatures. m—monoclinic phase of ZrO_2_, t—tetragonal phase of ZrO_2_.

The formation and evolution of the filament crystalline structure with increases in calcination temperature is shown in Figure 4. It can be clearly seen that nanofibers calcined at temperatures below 500 °C are amorphous. The increase in calcination temperature induces nanoparticle growth that results in the appearance of broad peaks in the XRD pattern at 500 °C. Observed reflections at 30.2°, 35.2°, 50.2° and 60.2° correspond to tetragonal (t-ZrO_2_) zirconia. The average t-ZrO_2_ grain size can be estimated as 8 nm from the characteristic peak at 30.2°, using the Scherrer equation.

The observed peaks of t-ZrO_2_ become sharper and narrower with a rise in calcination temperature to 800 °C, and reflections at 34.6°, 50.7°, and 59.3° are visualized. This indicates that the crystallinity is higher and the grain size is larger for zirconia nanofibers fabricated at higher calcination temperatures. The average t-ZrO_2_ grain size becomes 21 nm at 800 °C. At 800 °C, monoclinic (m-ZrO_2_) zirconia with characteristic peaks at 28.2° and 31.5° appears, and the t-ZrO_2_ content decreases from 100% to 67%. From the reflection at 28.2°, the average m-ZrO_2_ grain size is estimated to be about 25 nm via the Scherrer equation. A rise in calcination temperature from 800 to 1100 °C further stimulates m-ZrO_2 _content growth from 33% to 91%. The intensities of m-ZrO_2 _main peaks at 28.2° and 31.5° increase, and a large number of m-ZrO_2_ low-intensity peaks appear in the XRD pattern. The average m-ZrO_2_ grain size attains 70 nm, which is confirmed by SEM measurements (Figure 1c). At a calcination temperature of 1300 °C, t-ZrO_2_ completely transforms to m-ZrO_2_ as there are no t-ZrO_2_ peaks in the XRD pattern. Similar phase behavior of annealed electrospun composite fibers containing another ZrO_2_ precursor and another binding polymer was previously reported in [5,16,18]. Observed increases in ZrO_2_ grain size with rises in calcination temperature explain the above-mentioned simultaneous decreases in nanofiber specific surface area, since the latter are composed of ZrO_2_ grains.

In accordance with the obtained XRD data, the phase evolution of ZrO_2_ in ceramic nanofibers during their heat treatment includes the following stages: t-ZrO_2_ → t-ZrO_2_ + m-ZrO_2_ → m-ZrO_2_. According to Garvie’s theory, the tetragonal phase is thermodynamically more favorable than the monoclinic one for ZrO_2_ nanoparticles smaller than 30 nm [25]. With ZrO_2_ nanoparticle growth, the t-ZrO_2_ → m-ZrO_2_ transition probability increases. Ultimately, all t-ZrO_2_ nanoparticles become m-ZrO_2_ ones, since m-ZrO_2_ is stable up to 1170 °C [3]. That is observed in our case.

## 4. Conclusions

A novel combination of the ceramic precursor (zirconium acetylacetonate) and the binding polymer (polyacrylonitrile) for zirconia nanofiber fabrication via electrospinning was successfully tested. The produced ceramic filaments are real nanofibers, with a diameter of about 75 nm. It is revealed that a rise in calcination temperature from 500 to 1100 °C induces transformation of mesoporous t-ZrO_2_ nanofibers with a high specific surface area (102.3 m^2^/g) to non-porous m-ZrO_2_ nanofibers of almost the same diameter with a low value of specific surface area (8.3 m^2^/g). It is determined that the transition of electrospun zirconium acetylacetonate/polyacrylonitrile fibers to zirconia nanofibers is accompanied with the following stages of the ceramic precursor transformation: Zr(C_5_H_7_O_2_)_4_ → Zr(OH)(CH_3_COO)_3_ → ZrO(CH_3_COO)_2_ → t-ZrO_2_ → t-ZrO_2_+m-ZrO_2_ → m-ZrO_2_.

The t-ZrO_2_ nanofibers with high specific surface area prepared at 500 °C may be considered as perspective supports for heterogeneous catalysts, simultaneously enhancing the activity of the latter. For example, it is known that Ag and Rh supported on t-ZrO_2_ are far more active in the hydrogenation of carbon dioxide to methanol [26] and in the partial oxidation of methane to synthesis gas [27] than Ag and Rh correspondingly supported on m-ZrO_2_.
